# Supplementary material for: Deep learning-based stress detection for daily life use using single-channel EEG and GSR in a virtual reality interview paradigm
Source: PLoS One. 2024 Jul 3;19(7):e0305864. doi: 10.1371/journal.pone.0305864 (PMC11221693; doi:10.1371/journal.pone.0305864)
Supplement: Attachment — Submitted filename: Response to Reviewers.docx [file pone.0305864.s001.docx]

May 16, 2024

**Manuscript ID** PONE-D-24-09412

**Title**: Deep Learning-based Stress Detection for Daily Life Use Using Single-Channel EEG and GSR in a Virtual Reality Interview Paradigm

Dear Editor,

We thank you and the reviewers for your interest in our paper and for your comments, which have helped us improve the manuscript substantially. We have revised the paper in response to these comments, and we are glad to submit the revised manuscript for your consideration. We hope the revised manuscript will meet the requirements of your journal for publication.

Please do not hesitate to contact me if you have additional questions or issues regarding this paper.

We look forward to your final decision on this manuscript.

With best wishes,

Baek Hwan Cho, Ph.D.

Department of Biomedical Informatics, CHA University School of Medicine, CHA

University, Seongnam, Republic of Korea

Institute of Biomedical Informatics, School of Medicine, CHA University, Seongnam,

Republic of Korea

**Response to the academic editor**

**Journal requirements.**

When submitting your revision, we need you to address these additional requirements.

**Comment 1 (C1).** Please ensure that your manuscript meets PLOS ONE's style requirements, including those for file naming. The PLOS ONE style templates can be found at https://journals.plos.org/plosone/s/file?id=wjVg/PLOSOne_formatting_sample_main_body.pdf and https://journals.plos.org/plosone/s/file?id=ba62/PLOSOne_formatting_sample_title_authors_affiliations.pdf

**Response (R1).** Thank you for providing the specific journal guidelines. I reviewed them and ensured that the manuscript complied accordingly.

**C2.** Thank you for stating in your Funding Statement: "This work was supported by the Brain Convergence Research Program of the National Research Foundation (NRF) funded by the Korean government (MSIT) (No. NRF-2021M3E5D2A01022391)."

Please provide an amended statement that declares *all* the funding or sources of support (whether external or internal to your organization) received during this study, as detailed online in our guide for authors at http://journals.plos.org/plosone/s/submit-now. Please also include the statement “There was no additional external funding received for this study.” in your updated Funding Statement. Please include your amended Funding Statement within your cover letter. We will change the online submission form on your behalf.

**R2.** The funding statement has been revised as follows: "This work was supported by the Brain Convergence Research Program of the National Research Foundation (NRF) funded by the Korean government (MSIT) (No. NRF-2023R1A2C2003577). This is all the funding that was received for this study."

**C3.** We note that you have indicated that there are restrictions to data sharing for this study. PLOS only allows data to be available upon request if there are legal or ethical restrictions on sharing data publicly. For more information on unacceptable data access restrictions, please see <http://journals.plos.org/plosone/s/data-availability#loc-unacceptable-data-access-restrictions>.

Before we proceed with your manuscript, please address the following prompts:

a) If there are ethical or legal restrictions on sharing a de-identified data set, please explain them in detail (e.g., data contain potentially identifying or sensitive patient information, data are owned by a third-party organization, etc.) and who has imposed them (e.g., a Research Ethics Committee or Institutional Review Board, etc.). Please also provide contact information for a data access committee, ethics committee, or other institutional body to which data requests may be sent.

b) If there are no restrictions, please upload the minimal anonymized data set necessary to replicate your study findings to a stable, public repository and provide us with the relevant URLs, DOIs, or accession numbers. For a list of recommended repositories, please see https://journals.plos.org/plosone/s/recommended-repositories. You also have the option of uploading the data as Supporting Information files, but we would recommend depositing data directly to a data repository if possible. We will update your Data Availability statement on your behalf to reflect the information you provide.

**R3.** Thank you for your guidance on data sharing. According to the approved IRB, we have shared the participant data in a public repository. All the data acquired during the experiment has been uploaded to the following webpage for your review:

<https://github.com/MIHLabCHA/VR-Stress-Interview-Experiment-Data>

Please feel free to check it at your convenience.

**C4.** We note that Figures 1 and 2 in your submission contain copyrighted images. All PLOS content is published under the Creative Commons Attribution License (CC BY 4.0), which means that the manuscript, images, and Supporting Information files will be freely available online, and any third party is permitted to access, download, copy, distribute, and use these materials in any way, even commercially, with proper attribution. For more information, see our copyright guidelines: http://journals.plos.org/plosone/s/licenses-and-copyright.

We require you to either (1) present written permission from the copyright holder to publish these figures specifically under the CC BY 4.0 license, or (2) remove the figures from your submission:

a. You may seek permission from the original copyright holder of Figures 1 and 2 to publish the content specifically under the CC BY 4.0 license.
b. We recommend that you contact the original copyright holder with the Content Permission Form (http://journals.plos.org/plosone/s/file?id=7c09/content-permission-form.pdf) and the following text: “I request permission for the open-access journal PLOS ONE to publish XXX under the Creative Commons Attribution License (CCAL) CC BY 4.0 (http://creativecommons.org/licenses/by/4.0/). Please be aware that this license allows unrestricted use and distribution, even commercially, by third parties. Please reply and provide explicit written permission to publish XXX under a CC BY license and complete the attached form.” Please upload the completed Content Permission Form or other proof of granted permissions as an ""Other"" file with your submission. In the figure caption of the copyrighted figure, please include the following text: “Reprinted from [ref] under a CC BY license, with permission from [name of publisher], original copyright [original copyright year].” b. If you are unable to obtain permission from the original copyright holder to publish these figures under the CC BY 4.0 license or if the copyright holder’s requirements are incompatible with the CC BY 4.0 license, please either i) remove the figure or ii) supply a replacement figure that complies with the CC BY 4.0 license. Please check copyright information on all replacement figures and update the figure caption with source information. If applicable, please specify in the figure caption text when a figure is similar but not identical to the original image and is therefore for illustrative purposes only.

**R4.** Thank you for the detailed guidance regarding copyright issues. Regarding Figure 1, it serves to describe the experimental protocol, and I've determined that a captured photo image is unnecessary. Therefore, I have replaced it with a concise text description. For Figure 2, since it was uncertain whether the copyright pertains to the device or the electrode patch, I have obtained copyright permissions for both components as per your provided format and have attached them separately. **Response to reviewers**

**Reviewer 1.**

This manuscript describes the use of different deep learning models to identify stress and clam states using EEG and GSR signals. The manuscript is well written but the authors should address the following questions and comments?

**Comment 1 (C1).** Why is the learning rate of the multi-column model 10 times smaller than the single column models?

**Response (R1).** Thank you for your insightful question about the difference in learning rates between the multi-column and single-column models. The decision to adopt a lower learning rate for the multi-column model was primarily due to its architecture, which incorporates weight sharing from the pre-trained single-column models. Given that these weights are already partially trained, it is important to apply updates more cautiously. Therefore, we employed a lower learning rate to allow for subtle and precise adjustments, aiming to fine-tune the model without significant deviations from the established patterns. This cautious approach helps maintain the stability of the model’s performance across various datasets and minimizes the risk of overfitting.

2.4 CNN and transformer model architectures (page 6, Line 171-173)

“In contrast to the approach used with the single-column model, this specific learning rate was selected because the model had already been partially trained, necessitating careful fine-tuning during updates to optimize performance effectively.”

**C2.** In the current setting, the multi-column model was initialized from the optimized single column models. Does the performance vary significantly if the multi-column model was instead initialized with the same Image-Net weights and trained using the same learning rate as the single column models?

**R2.** Thank you for your comment. We also trained the multi-column model using ImageNet weights. Using the best-performing ResNet50 as a benchmark, the AUC was 0.917±0.031. This is slightly lower compared to the performance achieved with weights from the optimized single-column models. Generally, initializing with weights from the optimized single-column models yielded better results than using ImageNet weights.

Additionally, when we experimented with the multi-column model initialized from optimized single-column models and used a learning rate of 0.01, the AUC was 0.945±0.013. We maintained our previous strategy of multiplying the learning rate by 0.1 every 10 epochs. This approach resulted in performance that was slightly lower but generally similar to previous results.

2.4 CNN and transformer model architectures (page 6, Line 173-176)

“Furthermore, when comparing the multiple-column model initialized with the same learning rate of 0.01 as the single model or initialized with ImageNet weights, it generally showed slightly better performance (data not shown).”

**C3.** The authors used spectrograms to convert time-series input signal to a 2D image for the CNN training. Has the authors considered any other time-frequency analysis methods, especially the continuous wavelet transform (CWT)? CWT has seen great success in preparing 2D data for CNN-classifiers in many other fields, for example:

<https://doi.org/10.1121/1.3384871>

<https://doi.org/10.1115/IMECE2023-112407>

**R3.** Thank you very much for your comment. At the beginning of our research, we deliberated on how to convert measured biometric signals into images for training a CNN-based model. However, we had not considered the continuous wavelet transform (CWT). Your suggestion appears to be an excellent one, and we see significant potential in utilizing CWT in future research.

4. Discussion (page 13, Line 321-324)

“In addition, although this study exclusively utilized spectrograms for training our models, future investigations could also explore employing the continuous wavelet transform (CWT) to potentially enhance our ability to analyze and classify complex signal patterns.”

**C4.** The authors used a 5min averaging window to smooth out the predictions. Is this window length too much, considering that the interview session is only 10-15mins?

**R4.** Thank you very much for your comment. You raise a valid point. Indeed, the 5-minute window might appear excessive given the interview sessions last only 10-15 minutes. However, we determined that defining stress based on short-term changes could be problematic, as stress does not fluctuate within mere 30-second intervals. Using a longer smoothing window aids in accurately tracking stress levels, reflecting the sustained stress patterns typically observed in daily life. Thus, after testing 1, 3, and 5-minute windows, we found that the 5-minute window provided the best performance and accuracy for capturing long-term trends. Nonetheless, future research with longer interview sessions would be valuable to further refine this approach.

4. Discussion (page 11, Line 316-320)

“Although a-5minute window might seem excessive for 10-15minute sessions, our testing across 1, 3, and 5-minute intervals demonstrated that the 5-minute window effectively captures sustained stress patterns and minimizes misclassification. We recommend further studies with longer sessions to refine this method.”

**C5.** The training time of each model should be reported.

**R5.** Thank you for your comment. We focused on the models with the highest AUC scores to report their training times. For the single-column models, it took approximately 60 minutes to complete 5-fold training for both the GSR-trained ResNet-152 and the EEG-trained Inception-V3. As for the multi-column model, the ResNet-50 showed the best results and required about 90 minutes to train.

2.4 CNN and transformer model architectures (page 6, Line 176-178)

“Regarding the training duration, training the single-column model across 5 folds and 30 epochs took approximately 1 hour. In contrast, the multi-column model required about 1.5 hours to complete the same number of epochs.”

**Reviewer 2.**

The authors stimulated people's stress state through virtual interviews, assessed people's stress state using questionnaires, and modeled people's stress state through EEG signals and GSR signals. Demonstrated that neural networks can assess human stress levels from both models. Here are comments:

**Comment 1 (C1).** By using VAS questionnaire as the evaluation method. How often did the subjects be test by the VAS? What is the granularity of the labeling?

**Response (R1).** Thank you for your insightful comment. To better understand the frequency of the VAS assessments, please refer to Figure 1. The VAS was conducted four times in total, before and after each stress session. The VAS Rest assessed the stress levels immediately after a rest period, while VAS Session 1 and VAS Session 2 measured the stress scores during each of the VR interview sessions designed to induce stress. Finally, VAS Recovery was used to track changes in stress levels after the recovery period. The granularity of the labeling was achieved using a 10-cm visual analogue scale where participants marked their level of stress. These marks were then measured with a ruler to millimeter precision, so, for example, a mark at 4.2 cm on the scale would score a 4.2.

3.1 Biosignal characteristics on each session of experiments (page 7, Line 198-200)

“The granularity of the labeling was achieved using a 10-cm visual analogue scale where participants marked their level of stress. These marks were then measured with a ruler to millimeter precision, so, for example, a mark at 4.2 cm on the scale would score a 4.2. Measurements were taken a total of four times, before and after each session, as shown in Fig. 1.”

**C2.** GSR and EEG are used as features. How are they combined in the networks? Is normalization needed?

**R2.** Thank you for your insightful question. In our multi-column architecture, GSR and EEG data are combined by concatenating the feature maps. This method preserves the individual characteristics of each biometric signal while merging multiple inputs into a single vector for model training. This approach enables the model to effectively extract and integrate information from each type of data, thereby enhancing both learning and prediction outcomes. For normalization, we apply mean subtraction to each pixel value of the spectrogram image to ensure that no single modality contributes disproportionately more than another.

2.4 CNN and transformer model architectures (page 6, Line 164-166)

“For normalization, mean subtraction was applied to each pixel value in the spectrogram image to balance the contribution of each modality, preventing any one from disproportionately influencing the model's performance.”

**C3.** The last sentence of Section 2.2 is missing a period, and it seems like the sentence isn't complete.

**R3.** Thank you for pointing out our error. We appreciate your detailed review of our manuscript. The sentence has been corrected.

2.2 Biosignal processing (page 5, Line 134)

“Subsequently, the magnitude of each component was quantified.”

**C4.** Why are there fewer normal samples compared to stress samples? It should be easier to collect normal samples.

**R4.** Thank you for your valuable comment. Collecting normal samples proved more challenging than anticipated. To obtain biosignals in a stress-free state, participants first needed time to adapt to the unfamiliar environment and the biometric recording equipment. Despite providing thorough pre-experiment instructions, if the adaptation period was too lengthy, participants could become drowsy. Therefore, we iterated through multiple pilot study protocols to adjust the ideal time for participants to adapt without stress, aiming to accurately collect normal samples. This meticulous adjustment contributed to the relatively lower number of normal samples compared to stress samples.

**C5.** The dataset partitioning method should be introduced. If a patient appears in both the training and testing sets, it could introduce bias into the results.

**R5.** Thank you for your astute observation. To prevent data from the same subject from mixing in the training and testing sets, we partitioned the subjects into 5 folds on a subject-wise basis. Thus, each patient's data was assigned exclusively to either a training or a testing set, ensuring no overlap. Additionally, any data imbalances that arose during this process were addressed through cost-sensitive learning. We will provide further details on our dataset partitioning approach in our revised manuscript.

2.5 Evaluation of the model and experimental settings (page 6, Line 181-186)

“To prevent data from the same subject from mixing in the training and testing sets, we partitioned the dataset into five subsets on a subject-wise basis. Each subject's data was exclusively assigned to one of these subsets, with four subsets allocated for model training and the remaining one reserved for validation. This method ensures no overlap between training and testing data, thereby mitigating potential overestimation of model performance.”

**C6.** In Section 2.4, all models were pretrained on ImageNet. Why was this done? There's a significant difference between spectrogram and ImageNet images.

**R6.** Thank you for your observation. Although there are significant differences between the spectrogram and ImageNet datasets, the volume of data available from human subjects was relatively limited. Typically, applying transfer learning with proven visual models, such as those pretrained on ImageNet, often results in better performance and faster convergence when adapted to different types of data. This approach has proven effective for our dataset as well, yielding quicker and improved outcomes. We also experimented with training a single-column model from scratch; however, the best-performing GSR-trained ResNet-152 model yielded an AUC of only 0.847±0.070. This result was notably lower compared to models utilizing pretrained ImageNet weights.

2.4 CNN and transformer model architectures (page 5, Line 156-158)

“Each model was initialized using a pre-trained ImageNet model due to significantly lower performance when training from scratch (data not shown).”

**C7.** Section 2.4 and Figure 3 introduced the methods used, including the development of the multiple-column CNN model. However, the Vision Transformer does not belong to CNN. The author should clarify how it was incorporated.

**R7.** Thank you for pointing out our error. We acknowledge that the Vision Transformer is not a CNN model, and its inclusion was based on the convenience of discussing it within the context of image analysis. However, we recognize that this could lead to confusion among readers. We will revise this section to clearly distinguish the Vision Transformer from CNN models and clarify its integration into our methodology.

Comprehensive revision of expressions throughout the entire manuscript.

**C8.** Are hyperparameters such as batch size and learning rate the same across all models?

**R8.** Thank you for your comment. We identified the optimal hyperparameters, including batch size and learning rate, through a grid search. This process allowed us to determine the best-performing hyperparameters. Subsequently, we applied these settings uniformly across all models during training.

**C9.** Why was a 5-minute interval chosen for smoothing in Figure 5?

**R9.** Thank you for your insightful comment. We found that tracking stress based on shorter-term changes (such as within a 30-second window) could lead to inaccuracies, as stress levels typically do not fluctuate so rapidly. Our approach of using a longer window helps in better representing sustained stress patterns, which are more reflective of real-life scenarios. After experimenting with 1, 3, and 5-minute intervals, the 5-minute window proved most effective for capturing these longer-term trends in stress levels. Future studies involving extended session durations would indeed be beneficial in further optimizing our methodology.

4. Discussion (page 12, Line 316-320)

“Although a-5minute window might seem excessive for 10-15minute sessions, our testing across 1, 3, and 5-minute intervals demonstrated that the 5-minute window effectively captures sustained stress patterns and minimizes misclassification. We recommend further studies with longer sessions to refine this method.”

**C10.** AUC alone is not sufficient. Other metrics should be provided for comparison.

**R10.** Thank you for your comment. We have updated the model evaluation metrics, including AUC, and have summarized them in Table 1

**C11.** What is the purpose of Figure 7? What information can we gain from it? GradCAM operates in a two-dimensional space, but the original EEG and GSR signals are one-dimensional. Is this approach reasonable?

**R11.** Thank you for your valuable feedback. The practice of examining biosignals in the frequency domain, as opposed to the time domain where they are initially captured, has been extensively utilized in prior research in biosignal analysis. We, too, wished to observe the biosignal data we collected within the frequency domain. Particularly with GSR, we anticipated that its signal characteristics, which change rapidly under stress, would impact the frequency bands. Based on prior research and our experimental results, as presented in Figure 4, we hypothesized that there could be dominant frequency power shifts in the EEG, such as changes in the alpha and beta ratio within specific frequency bands. We speculated that these changes might have been leveraged by the artificial intelligence model during learning. Figure 7 aims to illustrate the differences in specific power changes in the frequency domain of each biosignal under stress conditions and to verify if the AI model indeed utilizes these variations for its analysis.

4. Discussion (page 13, Line 325-334)

“The method of analyzing biosignals in the frequency domain, as well as the time domain, has been widely used in previous research. Our study was informed by the knowledge that GSR signals change more rapidly under stress conditions and that, as seen in Figure 4, there are variations in the alpha/beta ratio. We anticipated differences in the frequency domain as a result. Based on these insights, we aimed to investigate whether our AI model would predominantly consider these differences in frequency power during its learning process. Despite applying Grad-CAM analysis, the results, as observed, did not show significant differences in the Hz regions monitored before and after stress. This observation suggests that prioritizing overall information rather than focusing on individual frequency bands may be crucial for the accurate classification of normal and stress states.”

**C12.** Data and code should be made publicly available.

**R12.** Thank you for your guidance on data sharing. According to the approved IRB, we have shared the participant data in a public repository. All the data acquired during the experiment has been uploaded to the following webpage for your review:

<https://github.com/MIHLabCHA/VR-Stress-Interview-Experiment-Data>

Additionally, we have shared both the data preprocessing MATLAB code and the deep learning model training Python code on the same repository.
